# Behavioural Nudges, Physico-Chemical Solutions, and Sensory Strategies to Reduce People’s Salt Consumption

**DOI:** 10.3390/foods11193092

**Published:** 2022-10-05

**Authors:** Charles Spence

**Affiliations:** Department of Experimental Psychology, New Radcliffe House, University of Oxford, Oxford OX2 6BW, UK; charles.spence@psy.ox.ac.uk

**Keywords:** salt-reduction strategies, gastrophysics, aroma, flavour, multisensory, congruency

## Abstract

This narrative historical review examines the wide range of approaches that has been trialled/suggested in order to reduce the consumption of salt. While sodium is an essential micronutrient, there is widespread evidence that high levels of consumption are leading to various negative health outcomes. This review summarises the evidence relating to the various approaches that have been put forward to date to help reduce salt consumption over the years, while also highlighting a number of important questions that remains for future research. Solutions to reducing salt consumption include everything from the gradual reduction in salt in foods through to the reduction in the number/size of holes in saltshakers (what one might consider a behavioural nudge). Physico-chemical solutions have included salt replacers, such as monosodium glutamate (MSG) through to the asymmetric distribution of salt in processed (e.g., layered) foods. A wide range of sensory approaches to modulating expected and perceived saltiness have also been suggested, including the use of salty aromas, as well as suggesting the use of colour cues, sonic seasoning, and even textural primes. It is currently unclear whether different salty aromas can be combined to increase odour-induced taste enhancement (OITE) effectiveness. In the years ahead, it will be interesting to assess how long such solutions remain effective, as well as whether different solutions can be combined to help reduce salt consumption without having to compromise on taste/flavour

## 1. Introduction

Salt is an essential mineral in the human diet and has long been used to help season and, thus, add flavour to food [1]. Since pre-history, salt has also played an important preserving function (e.g., in the case of salting fish and meat) [2,3]. It has been suggested that salt has occupied a place of culinary dominance across cultures for centuries [4]. According to Rachel Herz [5]: “We like the taste of salt innately because salt is a signal of protein in nature” (quoted in [6]). Others have argued, perhaps more persuasively, that the allure of salt taste is that it signals the presence of essential minerals instead [7]. Interestingly, however, research from van Dongen and colleagues has revealed that in a range of 50 commonly consumed foods, the presence of both salty and savoury tastes was independently correlated with the presence of both sodium and protein [8]. Humans need a small amount of salt to regulate fluid balance and help the nerves and muscles to function properly. The preference/need for salt is, however, not uniquely human. Famously, the monkeys at Koshima Inlet on an island off the coast of Japan salt sweet potatoes by washing them in sea water prior to consuming them [9,10]. Amazonian parrots flock to certain cliffs (mineral licks) to get the sodium and other nutrients they need, which is otherwise hard to come by in their diet [11,12,13]. It is commonly believed that providing salt licks for deer can give rise to improved antler quality [14]. Dogs, meanwhile, are fairly insensitive to the taste of salt, the argument being that meat, which would have featured heavily in their ancestral carnivorous diet, would naturally have been high in sodium [15,16].

Salt is both a very successful flavour enhancer and can also mask the taste of bitterness [17,18]. While salt is by no means the only basic tastant to perform such a function—umami, for example, has also been reported to suppress other tastes, while, at the same time enhancing the tastes of sweet and salt [19,20] and, therefore, it does a better job than any other tastant. However, while salt is undoubtedly an essential nutrient in the human diet, there is mounting evidence of the negative health consequences that are associated with the overconsumption of sodium. This, in turn, has led to growing calls for innovative salt-reduction strategies to help improve public health outcomes [21], strategies that go beyond merely taxing high-sodium foods as has been trialled in some countries [22]. 

Over the years, several different approaches have been proposed, from physico-structural solutions in the case of processed foods, through to various kinds of nudges, such as reducing the hole sizes in salt dispensers. Furthermore, everything from aromas to colours and from certain sounds to particular tactile/oral-somatosensory attributes have been shown to modulate expected, and often also perceived, saltiness, at least under carefully controlled laboratory conditions. The key question to be addressed in this review is how effective these various approaches to salt reduction might be in the long term in the real world and whether different approaches to salt reduction can be combined effectively. Finding long-term solutions to reduce salt intake is especially important given that salt warning and nutritional labels tend to have little effect [23,24]. In this narrative historical review, I summarize the evidence relating to the various approaches that have been put forward to date to help reduce salt consumption, while also highlighting a number of important questions that remain for future research. 

## 2. Negative Health Consequences of the Overconsumption of Salt

Despite the fact that small amounts of salt are essential for healthy nutrition, a growing body of robust scientific evidence has demonstrated that the overconsumption of salt in our diets is leading to a number of negative health consequences, including hypertension (i.e., high blood pressure), which increases the risk of heart disease and stroke [25], currently two of the leading causes of mortality. At the outset, though, it is important to note how the salt we consume comes not only from the foods that we choose to eat, but also from the sodium that is added at the table, either in the form of table salt (see [26,27] on the origins of table salt) or as a condiment, such as soy sauce, fish sauce, or Maggi-type seasoning [28]. Given the ready availability of table salt and salty condiments at the dining table, the danger is that simply reducing the salt levels in popular food products might well result in consumers adding salt (or salty condiments) to enhance the taste at the table [29]. Here it is intriguing to note that there have been some legislative efforts to remove salt from the dining tables of schools and kindergartens [30]. 

Bear in mind here that just a single tablespoon of mayonnaise contains around 11 g of fat, 100 cal, and 85 mg of sodium [31]. Some of the most shocking epidemiological evidence to have emerged recently suggests that those who regularly add salt to their food lower their life expectancy at 50 years of age by an average of 1.5 years for women and 2.3 years for men, relative to those who do not salt their food [25]. This was the result of a study of more than half a million participants from the UK biobank who were followed-up for a median of 9 years. While the current recommended daily intake of salt is 5–6 g [32,33], typical consumption figures are closer to double that, depending on the culture/country [34,35,36]. This contrasts markedly with the diets of our pre-agricultural ancestors, which may well have been low in salt, with estimated daily sodium intake of 768 mg, equivalent to a little under 2 g of salt [37].

While the rhetoric in the popular science press in recent years has been to blame the food industry for loading processed foods with sugar, salt, and fat [38,39], it should be noted that saltiness is not always a desirable characteristic. Indeed, food companies have been actively trying to suppress the salty taste of isotonic drinks to help make their beverages more palatable. At the same time, however, given the flavour-enhancing qualities of salt, reducing its presence in processed foods often results in an unacceptable loss in taste quality, leading to a backlash from consumers when the salt level of their favourite brands is reduced suddenly [40,41,42,43]. What is more, there are certain traditional foods, such as bread, where salt plays an essential structural function. In the latter case, the research shows that the addition of salt influences dough elasticity, improves taste, and also enhances the browning of the crust [44,45,46]. Salt is also a key ingredient in cheese making [47]. At the same time, however, there is a growing awareness of the exceptionally high levels of salt in some cheeses, such as Roquefort [48], where a 30 g serving contains more salt (1.06 g) than a small bag of crisps/potato chips. It has been estimated that more than 20–30% of dietary salt comes from meat and meat products [49] and that around 80% of salt consumed is hidden in processed, canteen, restaurant, and fast food [50]. Salt also provides an important role in helping to preserve meat products. Consequently, any attempt to reduce the salt levels may give rise to increased issues with food safety [51].

### What Determines the Preferred Salt Level?

There is some debate as to whether our salt preferences are hard-wired or learnt. For instance, one large global study (of more than 10,000 men and women over a 24 h period) found very similar levels of salt intake across a wide range of countries/cultures [52], thus, suggesting an innate preference. There is widespread evidence for an innate hunger for salt across many species, with salt hunger presumably helping to ensure a sufficient intake of sodium [53,54]. At the same time, however, other evidence shows that those on restricted sodium diets soon adapt to the reduced saltiness in their food [55]. In particular, Gary Beauchamp and colleagues conducted a study in which people put on a controlled, low-sodium diet showed taste adaptation. Specifically, within a period of one to two months, the amount of salt that the participants found optimal in soup or crackers declined by 40 or 50 percent. Put the other way, the more salt people eat, the more they crave it. The way in which people taste their food has also been shown to influence perceived saltiness [56]. Separately, there has been an ongoing debate in the food science literature about how best to measure salt thresholds [57,58]. Bear in mind here only how in perception threshold studies, participants are normally presented with water-based pure salt solutions, whereas most food products represent complex matrices, which may result in multi-factor perceptual interactions. One of the challenges here in a real-world context is that the way in which a food is labelled (or described) can also modulate rated saltiness as a function of expectations or even disconfirmed expectations [59,60].

Interestingly, infants only start to respond to the taste of salt at around six months of age [61,62]. At the other end of the age spectrum, the declining chemosensory abilities that have been extensively documented in the elderly can all too easily lead to unhealthy eating habits [63,64], as the latter increase their intake of salt and sugar to make up for their inability to taste these ingredients in food at lower concentrations [65,66]. According to Stevens and colleagues [67], older individuals may need to add as much as two- or three-times more salt to perceive the same intensity in a tomato soup as those who are younger. Worryingly, this figure was found to increase to twelve-times as much salt added for those older individuals who were on five or more medications, which turns out to be the majority of them [68]. Given the negative health consequences of the overconsumption of salt (e.g., hypertension), this likely represents a very serious issue and one that needs to be tackled by those hoping to optimise food delivery amongst the elderly. Note here that according to observational data from the Framingham study in North America, the lifetime risk of developing hypertension in those who are 55–65 years of age is 90% [69].

## 3. Behavioural Nudging to Modify Salt-Related Food Behaviours

In recent years, there has been growing interest in the potential use of a range of sensory nudges to influence consumer behaviour in the context of food and drink [70,71]. “Nudging” refers to the use of more-or-less subtle sensory contextual cues that are designed to encourage people to behave in ways that are likely to help in improving the health, social support welfare, sustainability, and/or happiness of the individual and/or the society in which they live. One strategy that has worked effectively to reduce salt levels in processed foods has involved what has been referred to as a ‘health-by-stealth’ approach [72]. Cereal companies have been gradually decreasing the amount of salt in their breakfast cereals. Overall, they have managed to reduce the salt content by up to 47% (between 1992 and 2015), without any noticeable customer backlash, simply by doing it so gradually that customers never became aware of any difference in the formulation [73]. Consequently, each successive reduction was essentially imperceptible to the consumer and, over the long term, salt levels have dropped substantially cf. [51,74,75,76,77].

It is important to note that behavioural nudges operate at different timescales, from the slow health-by-stealth approaches to reducing the salt content of, e.g., breakfast cereals, over the decades, through to the suggestion that the number and diameter of holes on saltshakers should be reduced [78,79,80]. The latter solution apparently significantly reduces the amount of salt that people add to their food, at least in the short term. Note that a similar approach has been suggested more recently in terms of reducing the use of fish sauce in university canteens serving noodles in Thailand. In particular, the results of one study indicated that a simple change in how fish sauce was served can reduce consumption. Specifically, serving fish sauce in a bowl with a spoon reduced the amount used per noodle bowl by 0.25 g, as compared to the typical situation in which fish sauce is served from a bottle [29]. Meanwhile, using a specially designed spoon with a hole induced a larger reduction of 0.58 g of fish sauce used per bowl. By contrast, the reduction in fish sauce usage associated with cognitive-/affectively oriented nudges failed to reach statistical significance. Similar solutions would presumably also help with soya sauce dispensers in Japan, given that it represents a significant source of dietary sodium for those living there [81]. According to Fukutome [82], soy sauce and other sauces containing soybean paste account for 15% of the amount of table sauce consumed in Thailand.

One of the key challenges, though, is that no other element plays such a flavour-enhancing function as salt. What is more, and as mentioned earlier, salt also plays a functional/structural role in many foods, such as bread and cheese/dairy, thus, making its effective replacement that much more challenging. Researchers have also considered the use of a range of other salt-reduction strategies. In the next section, I will review the evidence concerning various physico-chemical attempts to find salt replacers that are not derived from sodium and potential solutions associated with modifying the structural properties in processed foods.

## 4. Physico-Chemical Solutions of Salt Reduction While Maintaining Taste

There has long been interest in the possibility of finding stimuli that taste salty without relying on sodium. Molecular salt replacers, such as potassium chloride (KCl), iodized salt, etc., have been on the market for decades, often marketed as ‘low-sodium’ [83]. However, none of the other metal salts give rise to anything like as salty a taste as sodium. Furthermore, potassium chloride imparts a bitter taste if present in too high a proportion in foods [48,84]. More worryingly, an excess of potassium may cause hyperkalaemia (the name given to an abnormally high level of potassium in the blood leading to cardiac arrest), which is why many authorities do not recommend the use of potassium chloride as a salt substitute [85]. Others have been championing the replacement of sodium chloride with monosodium glutamate as an effective route to maintain taste/flavour, while reducing the amount of sodium consumed [86]. Note that MSG also gives rise to a somewhat salty taste [87]. Despite containing only one-third the amount of sodium (12.3 g/100 g) found in salt (39.3 g/100 g), it has been suggested that this makes MSG a particularly promising salt alternative in sodium-reduction strategies, though it is important to stress the independence of salty and umami as taste qualities, as stressed in ISO 3972. However, as will be mentioned later, some consumers show an aversion to ‘artificial’ flavour enhancers, as MSG is often labelled.

Separate to work on the development of salt replacers, a number of innovative physico-chemical food structural solutions to salt reduction have also emerged in recent years. Therefore, for example, it has been demonstrated that it is possible to maintain the perceived flavour of layered processed foods, such as lasagne, by asymmetrically distributing the salt through the food matrix rather than distributing it evenly [88,89], loading the salt taste in the first mouthful of other foods [90]. The evidence suggests that asymmetrically distributing the salt can give rise to enhanced (or maintained) taste perception while reducing the absolute quantity of salt. There may also be grounds for trying to target the salt to those parts of the oral cavity where taste receptors are more densely distributed [91,92].

Another promising approach to salt reduction involves the use of non-gustatory sensory stimuli in order to modulate the expected, and hopefully also the experienced, saltiness of foods. Therefore, for example, colours, aromas, textures, and even sonic stimuli have been shown to be associated with saltiness and can, under laboratory conditions at least, influence perceived saltiness.

## 5. Retronasal Enhancement of Saltiness Perception

One of the most popular/promising approaches to reducing salt, while, at the same time, maintaining taste, involves targeting the key role played by so-called salty aromas (see [93] for a recent review). The evidence from a growing body of empirical research demonstrates that those aromas that commonly co-occur with a salty taste in food (aromas that can be considered as congruent with a salty taste [94]) tend to take on the association with the co-occurring taste [95] and become more perceptually similar to the taste with which they commonly co-occur [96]. Therefore, for example, the odour of soy sauce and dried ham is classed as salty odour by those who are familiar with them. What is more, having become associated with a salty taste, these aromas, when added to foods give rise to odour-induced taste enhancement (OITE). This is where perceived saltiness is enhanced by the addition of odorants that are congruent (i.e., which commonly co-occur) with salty-tasting foods. At the same time, however, the presence of a specific taste (e.g., salty, umami, or sweet), has also been documented to enhance the associated food odour, no matter whether experienced orthonasally or retronasally [97,98,99].

Relevant to the theme of the present review, it has been widely suggested that OITE could be used as an effective strategy in order to enhance the perceived saltiness of reduced-salt food products. As highlighted in Table 1, many salty aromas that are clearly capable of giving rise to an OITE effect have been identified, there are several fundamental questions concerning OITE effects that remain to be addressed. 

Therefore, for example, one might wonder whether certain salty odorants are more effective than others at enhancing the perceived salty taste in food. It is currently also rather unclear whether different salty odorants can be combined to enhance their effectiveness. Recent findings also question whether heterogenous salt distribution can be combined effectively with OITE in realistic food substrates, such as hot flan [118]. The role of cultural differences in the OITE effects also constitutes an intriguing area for further study. To what extent does the aroma of specific local salty condiments come to take on enhanced OITE effects? Think here of Thai fish sauce in Thailand [29], soy sauce in Japan [81], or Maggi-type seasoning in a number of other countries [28]. Intriguingly, researchers have already started to probe the different representations of the qualities of soy sauces amongst consumers and chefs as a function of their sensory qualities cf. [119,120,121]. Another intriguing issue that has yet to be resolved is the relative importance of congruency to OITE effects (i.e., as distinct from the effects of perceptual similarity, see [94,96,99,122] on this subtle/intriguing distinction). Nevertheless, despite these outstanding issues, it is becoming increasingly clear that OITE represents a promising sensory approach to the maintenance of an acceptable taste/flavour profile in reduced-salt processed food products.

## 6. Visual Modulation of Saltiness Expectations and Perception

Beyond the olfactory contribution to salt perception, there has been much interest in the use of food colour to enhance perceived saltiness (e.g., in chicken stock/broth/bouillon [123,124,125]). In fact, a large body of research conducted over the last 90 years or so has demonstrated that colour cues in food and drink often exert a significant effect on taste thresholds [126], suprathreshold intensity ratings, and flavour-identification responses [127,128]. In an influential early psychophysical study, Maga found that adding colour (red, green, or yellow) to otherwise colourless salt solution had no impact over the salt taste detection threshold, despite the significant influence of adding colour on the threshold for sweet, bitter, and sour [126]. Other researchers though have reported that increasing the intensity of orange colour from a lighter to a darker colour increased saltiness expectation (based on visual observation) in the context of mayonnaise-dipping sauce [129,130]. Wongthahan and colleagues conducted a study of soy sauces (light, medium, and dark) using both regular users (consumers) and culinary chefs. Their results highlighted an association between brown colour intensity with saltiness expectation/perception [119].

One problem, though, when it comes to the use of colour to modulate salt perception is that salty foods come in all manner of colours (this is the argument put forward by Maga [126] to explain the null results on salt detection thresholds he reports) and, hence, it has been suggested that colour may be a less effective cue to saltiness perception that in the case of these colours associated with other tastes (e.g., consider the association between pinkish-red and sweetness; [131,132]; see also [133]). While early research tended to focus on the association between orangey-brown colour of savoury stocks and broth [29,123,124,134,135] and brown colour of condiments/seasonings, such as fish sauce and soy sauce [119], contemporary research on the cross-modal correspondences that exist between colour patches and taste have highlighted a robust association between the colours white and blue (either when presented individually or else together) and a salty taste [136,137,138,139]. While the link between salt and white may well relate to the typically white colour of salt crystals, the blue association may well be the result of packaging colour conventions in the marketplace. It should also be noted that the colours orange/brown were often not available to participants in the laboratory studies of colour-taste correspondences, hence, perhaps explaining why this pairing did not appear in the results that have been reported [140].

Nevertheless, taken together, the research that has been published to date demonstrates that despite the fact that multiple colours are associated with saltiness, there may be the opportunity to enhance perceived saltiness through the use of colour cues that help to set expectations of saltiness in the consumer (cf. [141]). Further, going beyond the use of colour, there may be fruitful opportunities to combine salty colours with salty aromas in order to enhance their combined effectiveness in conveying an impression of saltiness in reduced-salt food products (cf. [131,142]).

## 7. Sonic Seasoning to Modify Saltiness Perception

Background noise has been shown to suppress the perception of salt (and sweetness) in both food products and pure taste solutions [143,144,145]. At the same time, however, the presence of food consumption sounds that are typically associated with a salty taste (such as the sound of someone noisily eating potato chips) has been shown to increase perceived saltiness [146], though note the danger in triggering misophonia in some proportion of consumers on hearing such sounds [72]. Intriguingly, sonic seasoning is where music and soundscapes with particular sonic properties are found to accentuate the corresponding taste property [147,148,149,150,151]. According to research from Wang and colleagues, the sonic qualities that are most strongly associated with saltiness were a long decay time, high auditory roughness, and a regular rhythm. Meanwhile, in terms of emotional associations, saltiness was matched with negative valence, high arousal, and minor mode [151]. A few years ago, one Beijing café even went so far as to introduce sweet sonic seasoning so that they could reduce the sugar content in their drinks without having to compromise on taste [152]. In the future, it would not seem beyond the realms of possibility to use sonic seasoning to modulate perceived saltiness in food and drink.

## 8. Tactile/Haptic Modulation of Saltiness Perception

In terms of tactile/oral-somatosensory influences on saltiness perception, early research from Christensen demonstrated an impact of the viscosity in solutions on perceived saltiness and sweetness [153], though, in this case, the mechanism behind underlying the effect might be a cross-modal correspondence between viscosity and taste or perhaps a physicochemical impact of reduced OITE due to increased viscosity. Elsewhere, Van Rompay and Groothedde [154] demonstrated that perceived saltiness of potato chips could be enhanced through the use of rough surface texture design of a serving bowl (cf. [155,156,157,158,159]). Importantly, however, these saltiness-enhancement effects were only documented for medium and full-salt crisps but not for no-salt crisps.

## 9. Challenges to Salt Reduction: The Knowledge–Behaviour Gap and Self-Efficacy

Self-efficacy refers to the perceived ability of an individual to exert personal control [160]. The first of the four main constructs in the Knowledge Behaviour Gap model is knowledge, followed by acceptance, intention, and behaviour [161]. Self-efficacy has been shown to play a major role in the maintenance of health behaviours across a variety of health domains [162]. Most chronic diseases are rooted in lifestyle factors and enhancing knowledge is useful to the extent that it subsequently leads to the modification of people’s behaviour [163,164,165]. Intriguingly, dietary modification through enhancement of self-efficacy and knowledge was a principal area of focus of the FCP (Stanford Five-City Project) campaign with some success [166]. That said, a recent study among people living in Hong Kong highlighted a worrying disengagement with salt-reduction behaviour, such as rarely/never checking the sodium or salt content listed on the food label and rarely/never purchasing food labelled as containing low-salt or no-salt [167]. Meanwhile, nearly 90% of the participants in one Greek study did not know what the recommended daily salt intake was [168]. While 90% of the participants in another study conducted in the Australian state of Victoria were aware that excessive salt intake can cause health damage, more than 80% still acknowledged that they were eating “far too much” (i.e., more than the recommended daily intake), with less than half of the participants actively attempting to reduce their salt intake [169]. Importantly, for fundamental knowledge regarding the recommended daily intake, the primary food sources of high salt content and the differences between salt and sodium continues to be lacking, even amongst those living in high-income countries [170]. According to the results of a Chinese study, promoting better public knowledge, enhancing the public’s awareness of salt reduction, and encouraging more active salt-reduction behaviour can help to suppress the transition from normal blood pressure to hypertension [171]. Ultimately, researchers believe that the population’s knowledge, attitudes, and behaviours will affect their salt consumption and are thought to be adjustable and controllable intermediate factors over the short term [172].

## 10. Conclusions

The negative health consequences of the overconsumption of salt are becoming increasingly apparent [173,174,175]. This is despite the discrepancy between the WHO recommendations that people should keep their salt intake below 5 g per day (2000 mg/day of sodium) and their potassium intake above 3500 mg/day, and the dietary reference intakes from the National Academy of Sciences, Engineering, and Medicine in the USA that the adequate intake in adults is 3.75 g/day of salt (1500 mg/day of sodium), while the adequate potassium intake is 3400 mg/day in men and 2600 mg/day in women [176]. The dangers have, for example, been highlighted by the latest epidemiological research, showing that people who salt their food have a 1.5–2.3-year reduction in average life expectancy when compared to those who do not [25]. At the same time, however, the potential health benefits of reducing salt intake (e.g., in terms of lowering blood pressure) are also becoming increasingly well-established [177,178]. In fact, effective salt-reduction strategies have been rolled out in several countries in recent years [179] and these have provided learnings for those wanting to introduce effective public health strategies around salt reduction [180]. That said, while the major sources of dietary salt have now been identified [49,50,51,181,182], one of the challenges is that unless salt reduction occurs very gradually, it can lead to a negative consumer response in terms of impaired taste/flavour perception [41,183,184,185,186]. Numerous different solutions have been proposed, including behavioural nudges, such as reducing the number/size of holes in saltshakers [78,79,80]. In recent years, researchers have increasingly been attempting to take such suggested solutions from the laboratory to increasingly realistic contexts [187,188,189]. In one recent series of four controlled laboratory studies, five-holed saltshakers were shown to deliver around 34% of the salt of 17-holed saltshakers [186]. The question of which solutions are likely to have the greatest long-lasting effect, and separately whether the various factors can be combined, is, though, an important current area for research ([118,190]).

It is currently unclear the extent to which sensory modulations of perceived saltiness, such as via OITE effects, or the use of colour cues or sonic seasoning are capable of influencing neural activity in primary taste areas [191]. Relevant here, verbal labels/descriptions have been documented to modulate primary taste areas [192,193]. That said, a colour’s effect on consumer responses can sometimes trigger a response bias [128], while even visually presented shapes can at least under certain conditions influence taste thresholds, arguing that at least some of the cross-modal effects may have genuine perceptual consequences [194]. It would seem likely that such a cross-modal modulation of salt perception is likely to be more acceptable in the long term than the electric cutlery that has been invented by Japanese researchers [195]. Indeed, it is worth noting that the various salt-reduction strategies that have been studied over the years clearly differ markedly in their ease/practicality of implementation.

According to Liem and colleagues [185], a sodium reduction of up to 30% may be acceptable in processed foods if introduced gradually (i.e., over a period of 3 years) and that the reduction can be up to 50% as long as it is in parallel with the addition of a flavour-boosting ingredient, such as soy sauce or dried bonito. The addition of chilli can also be used to help spice-up foods, as can black pepper, etc., without the negative health consequences associated with the overconsumption of salt [196]. Others have proposed that reductions in salt (and sodium) can be mitigated with monosodium glutamate (MSG) in what they have termed the ‘salt flip’ [83,197,198]. MSG is the sodium salt of L-glutamic acid. It is the most abundant amino acid in nature, constituting up to 8% to 10% of most dietary proteins, either as free glutamate or bound to other amino acids. At the same time, however, consumer concerns with the consumption of MSG (often labelled as an ‘artificial’ taste or flavour enhancer), although misplaced, continue to limit the applicability of such an approach [199,200,201,202,203,204,205,206]. The lack of familiarity of many Western consumers with umami may not help either [207,208]. Finally, here, one might consider Nico Ladenis, the Greek-born restaurateur who was known for kicking customers out of his London restaurant should they be so foolish as to ask for the salt [209] (p. 215). Given the appeal of salt, which can all too easily turn to salt hunger [210], one might wonder whether such extreme responses will ultimately be needed in order to deal with the very serious problem of the overconsumption of salt, though given that it has been estimated that more than 70% of our salt consumption comes from food consumption outside the home (i.e., from ready meals and eating out) [211,212], it is clear that, ultimately, various other strategies are also going to be needed (see also [213]).

## Figures and Tables

**Table 1:** Chronological summary of published studies that have specifically studied the salt/umami taste-enhancing properties of volatile aromas. Adapted and updated from [93].

Study	Participants	Volatile Aroma	Taste Quality	Result	Comments
Murphy & Cain [100]	20 experienced p’s	Citral (citrus)	Sweet	Sig.	Aroma increased both congruent and incongruent taste intensity
			Salt	Sig.	
Frank & Byram [101]	E1: 20; E2: 20; E3: 18 untrained p’s	E1: Strawberry; E2: Peanut butter; E3: Strawberry	E1, E2: Sweet E3: Salt	Sig. n.s.	Only congruent strawberry aroma led to OITE in whipped cream base
Djordjevic et al. [102]	E1 (40 untrained p’s) E2 (same 40 p’s)	Strawberry & soy sauce	Sweet Salt	E1: Sig. E2: Sig.	Both actual and imagined odours enhanced congruent taste quality
Lawrence et al. [103]	59 untrained p’s	Range of salty food aromas including bacon and sardine	Salt	Sig.	7 salty aromas gave rise to significant enhancement in salt perception
Batenburg & van der Velden [104]	10 p’s & 2 trained panels of 10–12; Consumer panels	Chicken flavouring (inc. individual salty volatiles; e.g., sotolon)	Salt	Sig.	Untrained panel exhibited greater odour-induced salt enhancement in bouillons
Lawrence et al. [105]	27 untrained consumers	Comté cheese, sardine, & carrot	Salt	Sig.	Comté cheese & sardine aroma increased saltiness of model cheese
Nasri et al. [106]	64 untrained p’s	Sardine	Salt	Sig.	Sig. effect at low/medium salt levels, but no effect at high salt intensity
Nasri et al. [107]	61 untrained p’s	Sardine	Salt	Sig.	No effect of odour intensity on OITE
Seo et al. [108]	E1 (25 p’s); E2 (25 p’s)	Salty bacon & sweet strawberry	Salt Sweet	Sig. Sig.	Psychophysical & neuroimaging study
Manabe et al. [109]	70 p’s	Dried bonito stock	Umami	Sig.	Increased palatability of saltiness
Niimi et al. [110]	10 trained panellists	Cheese aroma mixture containing 10 volatiles	Umami Bitter	Sig. Sig.	Increasing OITE with increasing aroma intensity
Chokumnoyporn et al. [111]	10 panellists	Soy sauce	Salt	Sig.	Sig. effect on salty solutions above & below salt threshold
Emorine et al. [112]	Consumer panel (82)	Ham	Salt	Sig.	Sig. effect on salt perception
Syarifuddin et al. [113]	31 panellists	Sardine or butter	Salt/Fat	Sig.	Congruent aroma enhanced salt or fat
Kakutani et al. [114]	12 p’s	Soy sauce	Salt	Sig.	Retronasal odour after drinking, but not orthonasal odour before drinking, significantly increased salty taste
Onuma et al. [115]	E1 (12 p’s); E2 (20 p’s) E3 (12 p’s)	Soy sauce	Salt	Sig.	Saltiness enhancement demonstrated psychophysically & using neuroimaging
Manabe et al. [116]	E1 (75 p’s) E2 (75 p’s)	Soy sauce & 3-methyl-1-butanol	Salt	Sig.	Saltiness enhancement demonstrated & role of key compound identified
Sinding et al. [117]	13 untrained p’s	Beef stock	Salt	Sig.	Saltiness of reduced salt green pea soup

p’s—participants; Sig.—A significant enhancement on rated salty taste of the volatile was reported; n.s.—non-significant; OITE—odour-induced taste enhancement; inc.—including.

## Data Availability

No data is contained within the article.

## References

[1] Kaushik S., Kumar R., Kain P. (2018). Salt an Essential Nutrient: Advances in Understanding Salt Taste Detection Using Drosophila as a Model System. J. Exp. Neurosci..

[2] Roberts J. (2017). Salted and Cured: Savoring the Culture, Heritage, and Flavor of America’s Preserved Meats.

[3] Weller O., Dumitroaia G. (2005). The earliest salt production in the world: An early Neolithic exploitation in Poiana Slatinei-Lunca, Romania. Antiquity.

[4] Kurlansky M. (2003). Salt: A World History.

[5] Herz R. (2017). Why You Eat What You Eat: The Science Behind Our Relationship with Food.

[6] Jacewicz N. How Did Salt and Pepper Become the Mainstays of Western Cuisine. https://www.npr.org/sections/thesalt/2018/02/02/582477785/how-did-salt-and-pepper-become-the-soulmates-of-western-cuisine.

[7] Schulkin J. (1991). The allure of salt. Psychobiology.

[8] Van Dongen M.V., van der Berg M.C., Vink N., Kok F.J., De Graaf C. (2012). Taste–nutrient relationships in commonly consumed foods. Br. J. Nutr..

[9] Kawai M. (1965). Newly-acquired pre-cultural behavior of the natural troop of Japanese monkeys on Koshima islet. Primates.

[10] Matsuzawa T. (2015). Sweet-potato washing revisited: 50th anniversary of the Primates article. Primates.

[11] Emmons L.H., Stark N.M. (1979). Elemental Composition of a Natural Mineral Lick in Amazonia. Biotropica.

[12] Blair-West J.R., Coghlan J.P., Denton D.A., Nelson J.F., Orchard E., Scoggins B.A., Wright R.D., Myers K., Junqueira C.L. (1968). Physiological, morphological and behavioural adaptation to a sodium deficient environment by wild native Australian and introduced species of animals. Nature.

[13] Wiener J.G. (1975). Nutrient cycles, nutrient limitation and vertebrate populations. Biologist.

[14] Schultz S.R., Johnson M.K. (1992). Effects of supplemental mineral licks on white-tailed deer. Wildl. Soc. Bull..

[15] Coile C. Accounting for Taste: What Do Dogs Find Most Delicious?. https://www.akc.org/expert-advice/nutrition/accounting-taste-probing-mysteries-dogs-find-delicious/.

[16] Kitchell R.L. (1978). Taste perception and discrimination by the dog. Adv. Vet. Sci. Comp. Med..

[17] Breslin P.A.S., Beauchamp G.K. (1997). Salt enhances flavor by suppressing bitterness. Nature.

[18] Breslin P.A.S., Beauchamp G.K. (1995). Suppression of bitterness by sodium: Variation among bitter taste stimuli. Chem. Senses.

[19] Suwankanit C., Dermiki M., Kennedy O.B., Methven L. Umami: Suppressed by all other tastes but itself an enhancer of salty and sweet perception. Proceedings of the 10th Pangborn Sensory Science Symposium.

[20] Baines D., Brown M. (2016). Flavor enhancers: Characteristics and uses. Encyclopedia of Food and Health.

[21] Webster J., Dunford E., Huxley R., Li N., Nowson C.A., Neal B. (2009). The development of a national salt reduction strategy for Australia. Asia Pac. J. Clin. Nutr..

[22] Dodd R., Santos J.A., Tan M., Campbell N.R.C., Ni Mhurchu C., Cobb L., Jacobson M.F., He F.J., Trieu K., Osornprasop S. (2020). Effectiveness and feasibility of taxing salt and foods high in sodium: A systematic review of the evidence. Adv. Nutr. Int. Rev. J..

[23] Rojas-Rivas E., Antúnez L., Cuffia F., Otterbring T., Aschemann-Witzel J., Giménez A., Ares G. (2020). Time orientation and risk perception moderate the influence of sodium warnings on food choice: Implications for the design of communication campaigns. Appetite.

[24] Pavia W. (2015). New York peppers menus with salt warnings. The Times.

[25] Ma H., Xue Q., Wang X., Li X., Franco O.H., Li Y., Heianza Y., Manson J.E., Qi L. (2022). Adding salt to foods and hazard of premature mortality. Eur. Heart J..

[26] Lander N. (2012). The Art of the Restaurateur.

[27] Time (1982). A Brief History of Salt. Time.

[28] Spence C. (2018). The psychology of condiments: A review. Int. J. Gastron. Food Sci..

[29] andenbroucke J.P., von Elm E., Altman D.G., Gøtzsche P.C., Mulrow C.D., Pocock S.J., Poole C., Schlesselman J.J., Egger M., STROBE Initiative (2007). Strengthening the Reporting of Observational Studies in Epidemiology (STROBE): Explanation and elaboration. PLoS ONE.

[30] UK Government. Department of Education Guidance: School Food Standards Practical Guide (Updated 11 May 2022). https://www.gov.uk/government/publications/school-food-standards-resources-for-schools/school-food-standards-practical-guide.

[31] Patel A. (2013). Bad condiments: 15 unhealthy condiments include ketchup, BBQ sauce and mayonnaise. Huffington Post.

[32] World Health Organization (2003). Diet, Nutrition and the Prevention of Chronic Diseases.

[33] Food Safety Authority of Ireland (2005). Salt and Health: Review on the Scientific Evidence and Recommendations for Public Policy in Ireland.

[34] Durack E., Alonso-Gomez M., Wilkinson M.G. (2008). Salt: A review of its role in food science and public health. Curr. Nutr. Food Sci..

[35] Beer-Borst S., Costanza M.C., Pechère-Bertschi A., Morabia A. (2009). Twelve-year trends and correlates of dietary salt intakes for the general adult population of Geneva, Switzerland. Eur. J. Clin. Nutr..

[36] Brown I.J., Tzoulaki I., Candeias V., Elliott P. (2009). Salt intakes around the world: Implications for public health. Int. J. Epidemiol.

[37] Eaton S.B., Konner M.J. (1997). Paleolithic nutrition revisited: A twelve-year retrospective on its nature and implications. Eur. J. Clin. Nutr..

[38] Moss M. (2013). Salt, Sugar, Fat: How the Food Giants Hooked Us.

[39] Moss M. (2013). The Extraordinary Science of Addictive Junk Food. The New York Times.

[40] Tozer J. (2011). The great HP Sauce revolt: Online fury over salt cut that ‘ruins the taste’. Daily Mail Online.

[41] Charles D. (2012). The Paradox and Mystery of Our Taste for Salt. NPR.

[42] Kilcast D., den-Ridder C., Kilcast D., Angus F. (2007). Sensory issues in reducing salt in food products. Reducing Salt in Foods: Practical Strategies.

[43] Ainsworth P., Plunkett A., Kilcast D., Angus F. (2007). Reducing salt in snack products. Reducing Salt in Foods: Practical Strategies.

[44] Nahar N., Madzuki I., Izzah N., Ab Karim M., Ghazali H., Karim R. (2019). Bakery science of bread and the effect of salt reduction on quality: A review. Borneo J. Sci. Technol..

[45] Belz M.C.E., Ryan L.A.M., Arendt E.K. (2012). The impact of salt reduction in bread: A review. Crit. Rev. Food Sci. Nutr..

[46] Carcea M., Narducci V., Turfani V., Mellara F.A. (2020). Comprehensive study on the influence of sodium chloride on the technological quality parameters of soft wheat dough. Foods.

[47] Goy D., Häni J.-P., Piccinali P., Wehrmüller K., Jakob E., Fröhlich-Wyder M.-T. (2012). Salt and Its Significance in Cheese Making.

[48] Lever A.-M. (2012). ‘Unnecessary’ High Salt Levels in Cheese, Health Group Warns. BBC News.

[49] Wirth F. (1991). Reducing the fat and sodium content of meat products. What possibilities are there?. Fleischwirtsch.

[50] He F.J., MacGregor G.A. (2005). Salt in food. Lancet.

[51] Inguglia E.S., Zhang Z., Tiwari B.K., Kerry J.P., Burgess C. (2017). Salt reduction strategies in processed meat products—A review. Trends Food Sci. Technol..

[52] Intersalt Cooperative Research Group (1988). Intersalt: An international study of electrolyte excretion and blood pressure. Results for 24 hour urinary sodium and potassium excretion. Intersalt Cooperative Research Group. BMJ.

[53] Wolf G., Pfaffman C. (1969). Innate mechanisms for regulation of sodium intake. Olfaction and Taste.

[54] Denton D. (1982). The Hunger for Salt.

[55] Bertino M., Beauchamp G.K., Engelman K. (1982). Long-term reduction in dietary sodium alters the taste of salt. Am. J. Clin. Nutr..

[56] Jeon S.-Y., Lee E.-K., Kim K.-O. (2014). The perceived saltiness of soup affected by tasting protocols. Food Qual. Prefer..

[57] O’Mahoney M., Heintz C. (1981). Direct magnitude estimation of salt taste intensity with continuous correction for salivary adaptation. Chem. Senses.

[58] O’Mahony M., Kingsley L., Harji A., Davies M. (1976). What sensation signals the salt taste threshold?. Chem. Senses Flavor.

[59] Barrós-Loscertales A., González J., Pulvermüller F., Ventura-Campos N., Bustamante J.C., Costumero V., Ávila C. (2012). Reading salt activates gustatory brain regions: fMRI evidence for semantic grounding in a novel sensory modality. Cereb. Cortex.

[60] Yeomans M.R., Chambers L., Blumenthal H., Blake A. (2008). The role of expectancy in sensory and hedonic evaluation: The case of smoked salmon ice-cream. Food Qual. Prefer..

[61] Desor J.A., Maller O., Andrews K. (1975). Ingestive responses of human newborns to salty, sour, and bitter stimuli. J. Comp. Physiol. Psychol..

[62] Desor J.A., Greene L.S., Maller O. (1975). Preferences for sweet and salty in 9- to 15-year-old and adult humans. Science.

[63] Davis M.A., Randall E., Forthofer R.N., Lee E.S., Margen S. (1985). Living arrangements and dietary patterns of older adults in the United States. J. Gerontol..

[64] Hughes G., Bennett K.M., Hetherington M.M. (2004). Old and alone: Barriers to healthy eating in older men living on their own. Appetite.

[65] Schiffman S., Graham B. (2000). Taste and smell perception affect appetite and immunity in the elderly. Eur. J. Clin. Nutr..

[66] Ferris A.M., Duffy V.B. (1989). Effect of olfactory deficits on nutritional status: Does age predict persons at risk?. Ann. N. Y. Acad. Sci..

[67] Stevens J.C., Cain W.S., Demarque A., Ruthruff A.M. (1991). On the discrimination of missing ingredients: Aging and salt flavour. Appetite.

[68] Frank M.E., Hettinger T.P., Mott A.E. (1992). The sense of taste: Neurobiology, aging, and medication effects. Crit. Rev. Oral Biol. Med..

[69] Vasan R.S., Beiser A., Seshadri S., Larson M.G., Kannel W.B., D’Agostino R.B., Levy D. (2002). Residual lifetime risk for developing hypertension in middle-aged women and men: The Framingham Heart Study. JAMA.

[70] Seo H.-S. (2020). Sensory Nudges: The Influences of Environmental Contexts on Consumers’ Sensory Perception, Emotional Responses, and Behaviors toward Foods and Beverages. Foods.

[71] Thomas-Danguin T., Guichard E., Salles C. (2019). Cross-modal interactions as a strategy to enhance salty taste and to maintain liking of low-salt food: A review. Food Funct..

[72] Spence C. (2017). Gastrophysics: The New Science of Eating.

[73] Stuckey B. (2012). Taste What You’re Missing: The Passionate Eater’s Guide to Why Good Food Tastes Good.

[74] BBC News Kraft Cuts Dairylea Fat and Salt. http://news.bbc.co.uk/2/hi/business/6345205.stm.

[75] Pombo-Rodrigues S., Hashem K.M., He F.J., MacGregor G.A. (2017). Salt and sugars content of breakfast cereals in the UK from 1992 to 2015. Public Health Nutr..

[76] Ma Y., He F.J., Yin Y., Hashem K.M., MacGregor G.A. (2016). Gradual reduction of sugar in soft drinks without substitution as a strategy to reduce overweight, obesity, and type 2 diabetes: A modelling study. Lancet Diabetes Endocrinol..

[77] MacGregor G.A., Hashem K.M. (2014). Action on sugar—Lessons from UK salt reduction programme. Lancet.

[78] Greenfield H., Maples J., Wills R.B.H. (1983). Salting of food—A function of hole size and location of shakers. Nature.

[79] Greenfield H., Smith A.M., Wills R. (1984). Influence of multi-holed shakers on salting on food. Hum. Nutr. Appl. Nutr..

[80] Farleigh C., Shepherd R., Wharf S. (1990). The effect of manipulation of salt pot hole size on table salt use. Food Qual. Prefer.

[81] Lioe H.N., Selamat J., Yasuda M. (2010). Soy sauce and its umami taste: A link from the past to current situation. J. Food Sci..

[82] Fukutome N. Soy Sauces of Asia: Soy Sauce Usage in the Philippines, Thailand and Vietnam. https://www.kikkoman.co.jp/kiifc/foodculture/pdf_25/e_014_021.pdf.

[83] dos Santos B.A., Campagnol P.C.B., Morgano M.A., Pollonio M.A.R. (2014). Monosodium glutamate, disodium inosinate, disodium guanylate, lysine and taurine improve the sensory quality of fermented cooked sausages with 50% and 75% replacement of NaCl with KCl. Meat Sci..

[84] Colmenero F.J., Ayo M., Carballo J. (2005). Physicochemical properties of low sodium frankfurter with added walnut: Effect of transglutaminase combined with caseinate, KCl and dietary fibre as salt replacers. Meat Sci..

[85] John S.K., Rangan Y., Block C.A., Koff M.D. (2011). Life-threatening hyperkalemia from nutritional supplements: Uncommon or undiagnosed?. Am. J. Emerg. Med..

[86] Halim J., Bouzari A., Felder D., Guinard J. (2020). The *Salt Flip*: Sensory mitigation of salt (and sodium) reduction with monosodium glutamate (MSG) in “Better-for-You” foods. J. Food Sci..

[87] Yamaguchi S., Kimizuka A., Filler L.J., Garattini S., Kare M.R., Reynolds A.R., Wurtman R.J. (1979). Psychometric studies on the taste of monosodium glutamate. Glutamic Acid: Advances in Biochemistry and Physiology.

[88] Dijksterhuis G., Boucon C., Le Berre E. (2014). Increasing saltiness perception through perceptual constancy created by expectation. Food Qual. Prefer.

[89] Emorine M., Septier C., Thomas-Danguin T., Salles C. (2013). Heterogeneous salt distribution in hot snacks enhances saltiness without loss of acceptability. Food Res. Int..

[90] Dijksterhuis G.B., Le Berre E., Woods A.T. (2010). Food products with improved taste.

[91] Spence C. (2021). Analysing stereotypical food consumption behaviours: ‘This way up?’ Is there really a ‘right’ way to eat a biscuit?. Int. J. Food Des..

[92] Spence C. (2022). The tongue map and the spatial modulation of taste perception. Curr. Res. Food Sci..

[93] Spence C. (2021). Factors affecting odour-induced taste enhancement. Food Qual. Prefer.

[94] Schifferstein H.N.J., Verlegh P.W.J. (1996). The role of congruency and pleasantness in odor-induced taste enhancement. Acta Psychol..

[95] Stevenson R.J., Boakes R.A., Calvert G.A., Spence C., Stein B.E. (2004). Sweet and sour smells: Learned synaesthesia between the senses of taste and smell. The Handbook of Multisensory Processing.

[96] Spence C. (2022). Searching for perceptual similarity within, and between, the (chemical) senses. i-Perception.

[97] Linscott T.D., Lim J. (2016). Retronasal odor enhancement by salty and umami tastes. Food Qual. Prefer..

[98] Dalton P., Doolittle N., Nagata H., Breslin P. (2000). The merging of the senses: Integration of subthreshold taste and smell. Nat. Neurosci..

[99] Lim J., Fujimaru T., Linscott T.D. (2014). The role of congruency in taste–odor interactions. Food Qual. Prefer..

[100] Murphy C., Cain W.S. (1980). Taste and olfaction: Independence vs interaction. Physiol. Behav..

[101] Frank R.A., Byram J. (1988). Taste–smell interactions are tastant and odorant dependent. Chem. Senses.

[102] Djordjevic J., Zatorre R.J., Jones-Gotman M. (2004). Odor-induced changes in taste perception. Exp. Brain Res..

[103] Lawrence G., Salles C., Septier C., Busch J., Thomas-Danguin T. (2009). Odour–taste interactions: A way to enhance saltiness in low-salt content solutions. Food Qual. Prefer..

[104] Batenburg M., van der Velden R. (2011). Saltiness enhancement by savory aroma compounds. J. Food Sci..

[105] Lawrence G., Salles C., Palicki O., Septier C., Busch J., Thomas-Danguin T. (2011). Using cross-modal interactions to counterbalance salt reduction in solid foods. Int. Dairy J..

[106] Nasri N., Beno N., Septier C., Salles C., Thomas-Danguin T. (2011). Cross-modal interactions between taste and smell: Odour-induced saltiness enhancement depends on salt level. Food Qual. Prefer..

[107] Nasri N., Septier C., Beno N., Salles C., Thomas-Danguin T. (2013). Enhancing salty taste through odour–taste–taste interactions: Influence of odour intensity and salty tastants’ nature. Food Qual. Prefer..

[108] Seo H.-S., Iannilli E., Hummel C., Okazaki Y., Buschhüter D., Gerber J., Krammer G.E., van Lengerich B., Hummel T. (2011). A salty-congruent odor enhances saltiness: Functional magnetic resonance imaging study. Hum. Brain Mapp..

[109] Manabe M., Ishizaki S., Yamagishi U., Yoshioka T., Oginome N. (2014). Retronasal odor of dried bonito stock induces umami taste and improves the palatability of saltiness. J. Food Sci..

[110] Niimi J., Eddy A.I., Overington A.R., Heenan S.P., Silcock P., Bremer P.J., Delahunty C.M. (2013). Aroma–taste interactions between a model cheese aroma and five basic tastes in solution. Food Qual. Prefer..

[111] Chokumnoyporn N., Sriwattana S., Phimolsiripol Y., Torrico D.D., Prinyawiwatkul W. (2015). Soy sauce odour induces and enhances saltiness perception. Int. J. Food Sci. Technol..

[112] Emorine M., Septier C., Andriot I., Martin C., Salles C., Thomas-Danguin T. (2015). Combined heterogeneous distribution of salt and aroma in food enhances salt perception. Food Funct..

[113] Syarifuddin A., Septier C., Salles C., Thomas-Danguin T. (2016). Reducing salt and fat while maintaining taste: An approach on a model food system. Food Qual. Prefer..

[114] Kakutani Y., Narumi T., Kobayakawa T., Kawai T., Kusakabe Y., Kuneida S., Wada Y. (2019). Saltiness intensity enhancements by odor of soy sauce synchronized with breathing. Trans. Virtual Real. Soc. Jpn..

[115] Onuma T., Maruyama H., Sakai N. (2018). Enhancement of saltiness perception by monosodium glutamate taste and soy sauce odor: A near-infrared spectroscopy study. Chem. Senses.

[116] Manabe M., Sakaue R., Obata A. (2020). Contribution of the retronasal odor of soy sauce to salt reduction. J. Food Sci..

[117] Sinding C., Thibault H., Hummel T., Thomas-Danguin T. (2021). Odor-induced saltiness enhancement: Insights into the brain chronometry of flavor perception. Neuroscience.

[118] Emorine M., Septier C., Martin C., Cordelle S., Sémon E., Thomas-Danguin T., Salles C. (2021). Salt and aroma compound distributions influence flavour release and temporal perception while eating hot-served flans. Molecules.

[119] Wongthahan P., Sae-Eaw A., Prinyawiwatkul W. (2020). Sensory lexicon and relationships among brown colour, saltiness perception and sensory liking evaluated by regular users and culinary chefs: A case of soy sauces. Int. J. Food Sci. Technol..

[120] Boakes R., Hemberger H. (2012). Odour-modulation of taste ratings by chefs. Food Qual. Prefer..

[121] Flaherty T.J., Lim J. (2017). Individual differences in retronasal odor responsiveness: Effects of aging and concurrent taste. Chemosens. Percept..

[122] Lim J., Johnson M.B. (2012). The role of congruency in retronasal odor referral to the mouth. Chem. Senses.

[123] Gifford S.R., Clydesdale F.M. (1986). The psychophysical relationship between color and sodium chloride concentrations in model systems. J. Food Prot..

[124] Gifford S.R., Clydesdale F.M., Damon R.A. (1987). The psychophysical relationship between color and salt concentrations in chicken flavored broths. J. Sens. Stud..

[125] Chan M.M., Kane-Martinelli C. (1997). The effect of color on perceived flavor intensity and acceptance of foods by young adults and elderly adults. J. Am. Diet. Assoc..

[126] Maga J.A. (1974). Influence of color on taste thresholds. Chem. Senses.

[127] Wang Q.J., Spence C. (2019). Drinking through rosé-coloured glasses: Influence of wine colour on the perception of aroma and flavour in wine experts and novices. Food Res. Int..

[128] Spence C., Levitan C., Shankar M.U., Zampini M. (2010). Does food color influence taste and flavor perception in humans?. Chemosens. Percept..

[129] Sukkwai S., Chonpracha P., Kijroongrojana K., Prinyawiwatkul W. (2017). Influences of a natural colourant on colour and salty taste perception, liking, emotion and purchase intent: A case of mayonnaise-based dipping sauces. Int. J. Food Sci. Technol..

[130] Sukkwai S., Kijroongrojana K., Chonpracha P., Pujols K.D., Alonso-Marenco J.R., Ardoin R., Prinyawiwatkul W. (2018). Effects of colorant concentration and ‘natural colour’ or ‘sodium content’ claim on saltiness perception, consumer liking and emotion, and purchase intent of dipping sauces. Int. J. Food Sci. Technol..

[131] Spence C., Stafford L. Multisensory sweetness enhancement: Comparing olfaction and vision. Smell, Taste, Eat: The Role of the Chemical Senses in Eating Behaviour.

[132] Wang Q.J., Mielby L., Thybo A.K., Bertelsen A.S., Kidmose U., Spence C., Byrne D.V. (2019). Sweeter together? Assessing the combined influence of product-related and contextual factors on perceived sweetness of fruit beverages. J. Sens. Stud..

[133] Sugimori E., Kawasaki Y. (2022). Cross-modal correspondence between visual information and taste perception of bitter foods and drinks. Food Qual. Prefer..

[134] Ikeda K. (2002). New seasonings. Chem. Senses.

[135] Fateminia M., Ghotbabadi T.D., Azad K.M. (2020). Perceptions of the taste of colors in children and adults. Color Res. Appl..

[136] Wan X., Woods A.T., van Den Bosch J.J.F., McKenzie K.J., Velasco C., Spence C. (2014). Cross-cultural differences in crossmodal correspondences between basic tastes and visual features. Front. Psychol..

[137] Spence C., Wan X., Woods A., Velasco C., Deng J., Youssef J., DeRoy O. (2015). On tasty colours and colourful tastes? Assessing, explaining, and utilizing crossmodal correspondences between colours and basic tastes. Flavour.

[138] Woods A.T., Marmolejo-Ramos F., Velasco C., Spence C. (2016). Using single colors and color pairs to communicate basic tastes II: Foreground–background color combinations. i-Perception.

[139] Woods A.T., Spence C. (2016). Using single colors and color pairs to communicate basic tastes. i-Perception.

[140] Spence C., Levitan C.A. (2021). Explaining crossmodal correspondences between colours and tastes. i-Perception.

[141] Chonpracha P., Gao Y., Tuuri G., Prinyawiwatkul W. (2019). Possible sugar and calorie reduction by visual cues: A case of syrup added to brewed coffee. J. Food Sci..

[142] Wang Q.J., Mielby L.A., Junge J.Y., Bertelsen A.S., Kidmose U., Spence C., Byrne D.V. (2019). The Role of Intrinsic and Extrinsic Sensory Factors in Sweetness Perception of Food and Beverages: A Review. Foods.

[143] Ferber C., Cabanac M. (1987). Influence of noise on gustatory affective ratings and preference for sweet or salt. Appetite.

[144] Woods A.T., Poliakoff E., Lloyd D.M., Kuenzel J., Hodson R., Gonda H., Batchelor J., Dijksterhuis G.B., Thomas A. (2011). Effect of background noise on food perception. Food Qual. Prefer..

[145] Yan K.S., Dando R. (2015). A crossmodal role for audition in taste perception. J. Exp. Psychol. Hum. Percept. Perform..

[146] Seo H.-S., Hummel T. (2011). Auditory–olfactory integration: Congruent or pleasant sounds amplify odor pleasantness. Chem. Senses.

[147] Guetta R., Loui P. (2017). When music is salty: The crossmodal associations between sound and taste. PLoS ONE.

[148] Knoeferle K.M., Woods A., Käppler F., Spence C. (2015). That sounds sweet: Using cross-modal correspondences to communicate gustatory attributes. Psychol. Mark..

[149] Knöferle K., Spence C., Knoeferle K. (2012). Crossmodal correspondences between sounds and tastes. Psychon. Bull. Rev..

[150] Wang Q., Woods A., Spence C. (2015). “What’s your taste in music?” A comparison of the effectiveness of various soundscapes in evoking specific tastes. i-Perception.

[151] Wang Q.J., Keller S., Spence C. (2021). Metacognition and crossmodal correspondences between auditory attributes and saltiness in a large sample study. Multisens. Res..

[152] Blecken D. (2017). Hold the Sugar: A Chinese Café Brand is Offering Audio Sweeteners. Campaign.

[153] Christensen C.M. (1980). Effects of solution viscosity on perceived saltiness and sweetness. Percept. Psychophys..

[154] van Rompay T.J., Groothedde S. (2019). The taste of touch: Enhancing saltiness impressions through surface texture design. Food Qual. Prefer..

[155] Escobar F.B., Wang Q.J., Corredor A., Velasco C. (2022). The taste of visual textures. Food Qual. Prefer..

[156] Seo H.-S., Iannilli E., Hummel C., Okazaki Y., Buschhüter D., Gerber J., Krammer G.E., Seo H.-S., Arshamian A., Schemmer K. (2010). Cross-modal integration between odors and abstract symbols. Neurosci. Lett..

[157] Juravle G., Olari E.-L., Spence C. (2022). A taste for beauty: On the expected taste, hardness, texture, and temperature of geometric shapes. i-Perception.

[158] Spence C., Corujo A., Youssef J. (2019). Cotton candy: A gastrophysical investigation. Int. J. Gastron. Food Sci..

[159] Pikler J. (1922). Schriften zur Anspassungstheorie des Empfindungsvorganges.

[160] Bandura A. (1977). Social Learning Theory.

[161] Stibe A., Krüger N., Behne A., Awan I., Younas M., Poniszewska-Marańda A. (2022). Knowledge behavior gap model: An application for technology acceptance. Mobile Web and Intelligent Information Systems. MobiWIS 2022. Lecture Notes in Computer Science, 13475.

[162] Maibach E., Murphy D.A. (1995). Self-efficacy in health promotion research and practice: Conceptualization and measurement. Health Educ. Res..

[163] National Research Council. Committee on Diet and Health (1989). Diet and Health: Implications for Reducing Chronic Disease Risk.

[164] Rimal R.N. (2000). Closing the knowledge-behavior gap in health promotion: The mediating role of self-efficacy. Health Commun..

[165] Tinsley B.J. (1992). Multiple influences on the acquisition and socialization of children’s health attitudes and behavior: An integrative review. Child Dev..

[166] Farquhar J.W., Fortmann S.P., Flora J.A., Taylor B., Haskell W.L., Williams P.T., Maccoby N., Wood P.D. (1990). Effects of community-wide education on cardiovascular disease risk factors: The Stanford five-city project. J. Am. Med. Assoc..

[167] Cheung J., Neyle D., Chow P.P.K. (2021). Current knowledge and behavior towards salt reduction among Hong Kong citizens: A cross–sectional survey. Int. J. Environ. Res. Public Health.

[168] Marakis G., Tsigarida E., Mila S., Panagiotakos D.B. (2014). Knowledge, attitudes and behavior of Greek adults towards salt consumption: A Hellenic food authority project. Public Health Nutr..

[169] Grimes C.A., Kelley S.J., Stanley S., Bolam B., Webster J., Khokhar D., Nowson C.A. (2017). Knowledge, attitudes and behaviors related to dietary salt among adults in the state of Victoria, Australia 2015. BMC Public Health.

[170] Bhana N., Utter J., Eyles H. (2018). Knowledge, attitudes and behaviours related to dietary salt intake in high-income countries: A systematic review. Curr. Nutr. Rep..

[171] Du X., Fang L., Xu J., Chen X., Bai Y., Wu J., Wu L., Zhong J. (2022). The association of knowledge, attitudes and behaviors related to salt with 24-h urinary sodium, potassium excretion and hypertensive status. Sci. Rep..

[172] Zandstra E.H., Lion R., Newson R.S. (2016). Salt reduction: Moving from consumer awareness to action. Food Qual. Prefer..

[173] Santos J.A., Sparks E., Thout S.R., McKenzie B., Trieu K., Hoek A., Johnson C., McLean R., Arcand J., Campbell N.R.C. (2019). The science of salt: A global review on changes in sodium levels in foods. J. Clin. Hypertens..

[174] Powles J., Fahimi S., Micha R., Khatibzadeh S., Shi P., Ezzati M., Engell R.E., Lim S.S., Danaei G., Mozaffarian D. (2013). Global, regional and national sodium intakes in 1990 and 2010: A systematic analysis of 24 h urinary sodium excretion and dietary surveys worldwide. BMJ Open.

[175] World Health Organization (2012). Guideline: Sodium Intake for Adults and Children.

[176] Oria M., Harrison M., Stallings V.A. (2019). Dietary Reference Intakes for Sodium and Potassium.

[177] He F.J., Tan M., Ma Y., MacGregor G.A. (2020). Salt reduction to prevent hypertension and cardiovascular disease: JACC state-of-the-art review. J. Am. Coll. Cardiol..

[178] He F., Li J., MacGregor G. (2013). Effect of longer term modest salt reduction on blood pressure: Cochrane systematic review and meta-analysis of randomised trials. BMJ.

[179] He F.J., Brinsden H.C., MacGregor G.A. (2014). Salt reduction in the United Kingdom: A successful experiment in public health. J. Hum. Hypertens..

[180] Winkler J.T. (2014). Nutritional reformulation: The unobtrusive strategy. Food Sci. Technol..

[181] Johnson C.M., Angell S.Y., Lederer A., Dumanovsky T., Huang C., Bassett M.T., Silver L.D. (2010). Sodium content of lunchtime fast food purchases at major US chains. Arch. Intern. Med..

[182] James W., Ralph A., Sanchez-Castillo C. (1987). The dominance of salt in manufactured food in the sodium intake of affluent societies. Lancet.

[183] Beauchamp G.K., Bertino M., Moran M. (1982). Sodium regulation: Sensory aspects. J. Am. Diet. Assoc..

[184] Hoppu U., Hopia A., Pohjanheimo T., Rotola-Pukkila M., Mäkinen S., Pihlanto A., Sandell M. (2017). Effect of salt reduction on consumer acceptance and sensory quality of food. Foods.

[185] Liem D.G., Miremadi F., Keast R.S.J. (2011). Reducing Sodium in Foods: The Effect on Flavor. Nutrients.

[186] Busch J., Feunekes G., Hauer B., den Hoed W. (2010). Salt reduction and the consumer perspective. New Food.

[187] Goffe L., Wrieden W., Penn L., Hillier-Brown F., Lake A.A., Araújo-Soares V., Summerbell C., White M., Adamson A.J., Adams J. (2016). Reducing the Salt Added to Takeaway Food: Within-Subjects Comparison of Salt Delivered by Five and 17 Holed Salt Shakers in Controlled Conditions. PLoS ONE.

[188] Goffe L., Hillier-Brown F., Doherty A., Wrieden W., Lake A.A., Araujo-Soares V., Summerbell C., White M., Adamson A.J., Adams J. (2016). Comparison of sodium content of meals served by independent takeaways using standard versus reduced holed salt shakers: Cross-sectional study. Int. J. Behav. Nutr. Phys. Act..

[189] Villinger K., Wahl D.R., Engel K., Renner B. (2021). Nudging sugar portions: A real-world experiment. BMC Nutr..

[190] Spence C. (2022). Why should vanilla be the most liked smell?. Nature Food.

[191] Nakamura Y., Goto T.K., Tokumori K., Yoshiura T., Kobayashi K., Nakamura Y., Honda H., Ninomiya Y., Yoshiura K. (2012). The temporal change in the cortical activations due to salty and sweet tastes in humans: fMRI and time-intensity sensory evaluation. NeuroReport.

[192] Woods A.T., Lloyd D.M., Kuenzel J., Poliakoff E., Dijksterhuis G.B., Thomas A. (2011). Expected taste intensity affects response to sweet drinks in primary taste cortex. NeuroReport.

[193] Nitschke J.B., Dixon G.E., Sarinopoulos I., Short S., Cohen J.D., E Smith E., Kosslyn S.M., Rose R.M., Davidson R. (2006). Altering expectancy dampens neural response to aversive taste in primary taste cortex. Nat. Neurosci..

[194] Liang P., Roy S., Chen M.-L., Zhang G.-H. (2013). Visual influence of shapes and semantic familiarity on human sweet sensitivity. Behav. Brain Res..

[195] Bolton A. (2016). No Salt, No Problem! Japanese Electro Fork Zaps Flavour into Your Mouth. CNet.

[196] Spence C. Gastrophysics: The psychology of herbs and spices. Proceedings of the Oxford Symposium on Food and Cookery.

[197] Hartley I.E., Liem D.G., Keast R.S. (2020). Females’ ability to discriminate MSG from NaCl influences perceived intensity but not liking of MSG added vegetable broths. J. Food Sci..

[198] Chi S., Chen T. (1992). Predicting optimum monosodium glutamate and sodium chloride concentrations in chicken broth as affected by spice addition. J. Food Process. Preserv..

[199] Maluly H.D.B., Arisseto-Bragotto A.P., Reyes F.G.R. (2017). Monosodium glutamate as a tool to reduce sodium in foodstuffs: Technological and safety aspects. Food Sci. Nutr..

[200] Wang S., Adhikari K. (2018). Consumer perceptions and other influencing factors about monosodium glutamate in the United States. J. Sens. Stud..

[201] Schaumburg H.H., Byck R., Gerstl R., Mashman J.H. (1969). Monosodium L-Glutamate: Its Pharmacology and Role in the Chinese Restaurant Syndrome. Science.

[202] Prescott J., Young A. (2002). Does information about MSG (monosodium glutamate) content influence consumer ratings of soups with and without added MSG?. Appetite.

[203] Halpern B.P. (2002). What’s in a name? Are MSG and umami the same?. Chem. Senses.

[204] Zautcke J.L., Schwartz J.A., Mueller E.J. (1986). Chinese Restaurant Syndrome: A review. Ann. Emerg. Med..

[205] Nierenberg A. (2020). The campaign to redefine ‘Chinese Restaurant Syndrome. The New York Times.

[206] McPhee S. (2022). Colonel Sanders’ top secret: Uproar after the crucial ingredient in KFC seasoning is exposed—And it’s left fried-chicken lovers SHOCKED. Daily Mail Online.

[207] Kobayashi C., Kennedy L.M., Halpern B.P. (2006). Experience-induced changes in taste identification of monosodium glutamate (MSG) are reversible. Chem. Senses.

[208] Kurihara K. (2015). Umami the fifth basic taste: History of studies on receptor mechanisms and role as a food flavor. BioMed Res. Int..

[209] Sitwell W. (2020). The Restaurant: A history of Eating Out.

[210] Schulkin J. (1991). Sodium Hunger: The Search for a Salty Taste.

[211] Harnack L.J., Cogswell M.E., Shikany J.M., Gardner C.D., Gillespie C., Loria C.M., Zhou X., Yuan K., Steffen L.M. (2017). Sources of Sodium in US Adults From 3 Geographic Regions. Circulation.

[212] Scourboutakos M.J., Semnani-Azad Z., L’Abbe M.R. (2013). Restaurant meals: Almost a full day’s worth of calories, fats, and sodium. JAMA Int. Med..

[213] Kolata G. (2017). Why everything we know about salt may be wrong. The New York Times.

